# Association of the cardiometabolic index with sarcopenia among U.S. adults: NHANES 2011–2018 findings

**DOI:** 10.1371/journal.pone.0323905

**Published:** 2025-05-15

**Authors:** Qing Zhao, Yue Xu, Xiaotian Chen

**Affiliations:** Department of Clinical Nutrition, Nanjing Drum Tower Hospital, Affiliated Hospital of Nanjing University Medical School, Nanjing, Jiangsu, China; McMaster University, CANADA

## Abstract

**Background:**

The cardiometabolic index (CMI), initially devised as a diagnostic tool for diabetes mellitus, has evolved into a composite biomarker for evaluating metabolic syndrome and cardiovascular disease risk. In order to shed light on any possible interactions between sarcopenia and CMI, this study will look at the relationship between the two.

**Methods and results:**

Data from the 2011–2018 National Health and Nutrition Examination Survey (NHANES) were analyzed to investigate the possible link between sarcopenia and CMI. Among 3,185 eligible participants, the weighted prevalence of sarcopenia was 7.84%. A significant positive association emerged between CMI and sarcopenia risk, with each unit increase in CMI was linked with a 12% greater risk of sarcopenia in the fully adjusted model (OR: 1.12; 95% CI: 1.01–1.26). Moreover, dose-response relationships were evident across CMI tertiles (*P* for trend < 0.05). Subgroup analyses and interaction tests indicated that the positive correlation between CMI and the risk of sarcopenia differs significantly across subgroups defined by education level, sedentary time and CVD status (all *P* for interaction < 0.05).

**Conclusions:**

Our findings demonstrate a robust association between elevated CMI levels and increased sarcopenia risk, suggesting CMI’s potential utility as a clinical biomarker for sarcopenia risk surveillance. To confirm these results and demonstrate causality, more research is required.

## Introduction

Sarcopenia, characterized by the progressive loss of skeletal muscle mass and function, predominantly affects older adults and is linked to frailty, falls, fractures, and increased mortality [1–4]. Its prevalence in elderly populations ranges from 5% to 50%, depending on diagnostic criteria [3,4]. In addition to its clinical consequences, sarcopenia imposes substantial healthcare burdens due to treatment costs and long-term care needs [5,6]. Identifying modifiable risk factors is critical for mitigating this growing public health challenge.

The Cardiometabolic Index (CMI), calculated as a combination of triglycerides (TG), high-density lipoprotein cholesterol (HDL-C), waist circumference (WC), and height, reflects central obesity and dyslipidemia [7,8]. CMI predicts metabolic diseases such as diabetes and cardiovascular disorders [9–12], but its association with sarcopenia remains unexplored. This gap is notable, given emerging evidence linking lipid metabolism to muscle health. For instance, elevated triglycerides and cholesterol correlate with reduced muscle mass [13,14], while dysregulated lipid profiles in sarcopenia suggest mitochondrial dysfunction and impaired energy metabolism [15,16]. Mechanistic evidence suggests that visceral adiposity, reflected by waist circumference, drives chronic systemic inflammation and insulin resistance, inhibiting muscle protein synthesis through dysregulated anabolic signalling pathways [17]. Furthermore, elevated triglyceride levels contribute to lipotoxicity by impairing mitochondrial integrity in skeletal muscle tissue, resulting in mitochondrial dysfunction and heightened oxidative stress, which collectively exacerbate muscle catabolism [18].

Given these associations, this study aims to investigate the potential of the CMI as a novel biomarker for sarcopenia risk by analyzing nationally representative data from the National Health and Nutrition Examination Survey (NHANES).

## Methods and materials

### Study population

This cross-sectional study utilized data from NHANES, a population-based survey conducted by the National Center for Health Statistics (NCHS), to assess the health and nutritional status of the U.S. population. NHANES employed a complex, stratified, multistage probability sampling design to obtain a representative sample of the non-institutionalized U.S. population.

**Fig 1:**
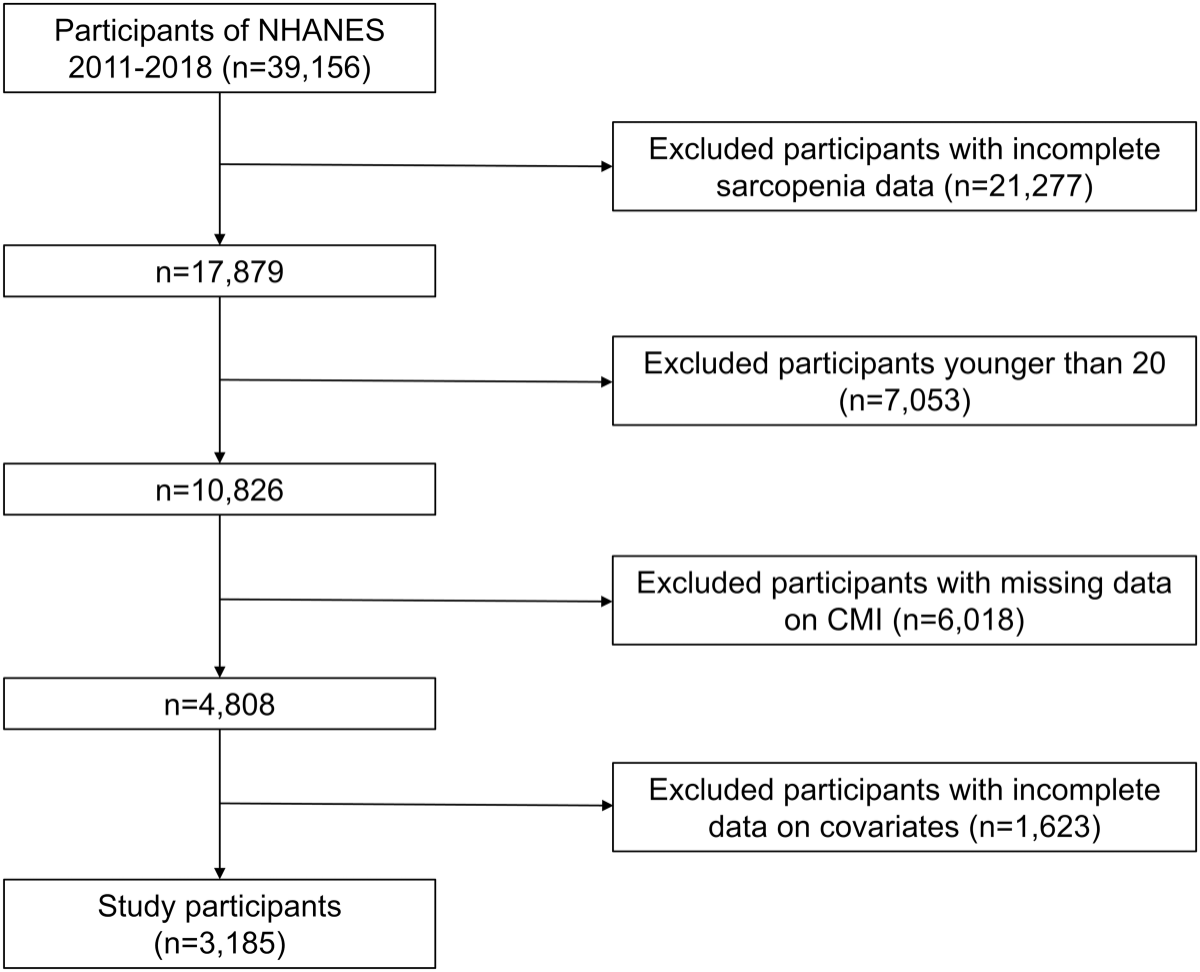
Flow chart of the study population.

We analyzed data from four NHANES cycles (2011–2018), comprising 39,156 individuals. Of these, 35,947 subjects were excluded for various reasons, including incomplete sarcopenia data (n = 21,277), age below 20 years (n = 7,053), missing CMI data (n = 6,018), and absence of covariate data (n = 1,623). Fig 1 illustrates the selection procedure. Ultimately, 3,185 individuals were included in the analysis. All NHANES participants provided written informed consent.

### Definition of sarcopenia and CMI

Sarcopenia in this study was assessed by measuring appendicular skeletal muscle mass (ASM) through dual-energy X-ray absorptiometry. ASM, representing the total lean tissue mass in the limbs, served as the primary indicator. The definition of sarcopenia followed the criteria established by the Foundation for the National Institutes of Health (FNIH) Sarcopenia Project [19]. Specifically, sarcopenia was diagnosed when the men’s and women’s sarcopenia index, calculated by dividing ASM by BMI, were less than 0.789 and 0.512, respectively.

The CMI, introduced by Wakabayashi et al. in 2015, has been validated as a reliable tool for assessing the risk of diabetes and other metabolic disorders [7]. The waist-to-height ratio (WHtR) is derived by dividing WC by height, and the specific formulas for calculating CMI are as follows:


CMI=TG(mmol/L)/HDL−C(mmol/L)×WHtR

### Covariates

Based on previous literature [20–23], this study incorporated a range of demographic variables, dietary factors, anthropometric measurements, physical activity, comorbidities, and laboratory parameters. Demographic variables comprised standard personal information and the poverty–income ratio (PIR), categorized into three categories: 1.3–3.5, ≥ 3.5, and <1.3. Two 24-hour dietary recalls were averaged out to determine total energy consumption. Anthropometric measurements included WC and body mass index (BMI). Physical activity data were obtained through a physical activity questionnaire. To standardize the physical activity data across periods, we included minutes of sedentary activity and vigorous work activity status. Sedentary time was further grouped into <3 hours, 3–6 hours, and ≥6 hours. Comorbidities assessed included hypertension, diabetes, and cardiovascular disease (CVD).

Laboratory parameters measured included HDL-c (mmol/L), TG (mmol/L), serum creatinine (SCR, umol/L), total cholesterol (TC, mmol/L), aspartate transaminase (AST, U/L); blood urea nitrogen (BUN, mmol/L), alanine transaminase (ALT, U/L), and serum uric acid (SUA, µmol/L). Biochemical markers related to mitochondrial metabolism were also included as covariates, such as creatine phosphokinase (CPK, IU/L) and serum iron (µmol/L). CPK is associated with mitochondrial function in cases of muscle injury or metabolic abnormalities [24], while serum iron levels are involved in the function of mitochondrial electron transport chain complexes [25]. Additionally, white blood cell counts were used as a marker of chronic inflammation. Detailed definitions of these variables are provided in the S1 Table.

### Statistical analysis

According to the recommendations from the NHANES website, NHANES employs a sophisticated sampling strategy to guarantee that the information gathered represents the whole country. Therefore, we incorporated sampling weights into our investigation, and the sample weight was calculated as 1/4 * WTDRD1. Continuous variables are reported as weighted medians and interquartile ranges (IQR), while categorical variables are shown as weighted percentages. The three CMI groups’ variable characteristics were compared using a weighted chi-square test and linear regression model. Univariate regression analysis was used to explore variables associated with sarcopenia. The weighted logistic regression analysis results, which assessed the relationship between sarcopenia and CMI, were shown as odds ratios (ORs) and 95% confidence intervals (CIs). In the multivariate analysis, the variance inflation factor (VIF) was used to assess multicollinearity, considering the interrelationships between the variables (S2 Table). Then, we developed several models to account for any confounding variables that might affect the results: Model 1 included all variables and did not adjust for confounders; Age, sex, and race/ethnicity adjustments were included in Model 2; and Model 3 involved further adjusted in light of Model 2 by including education level, PIR, marital status, BMI, hypertension, CVD, energy intake, diabetes, sedentary time, vigorous activity, smoking status, TC, ALT, AST, BUN, SCR, CPK, SUA, serum iron, and white blood cell. We then converted the continuous CMI scores into tertiles to explore trend tests (*P* for trend). For the sake of examining the linear association between CMI and sarcopenia, we used smooth curve fittings. Furthermore, subgroup analyses and interaction tests were conducted based on sex, education level, PIR, BMI, sedentary time, vigorous activity, and comorbidities (hypertension, diabetes, and CVD). All statistical analyses were performed using R (version 3.4.3) and EmpowerStats (http://www.empowerstats.com). Statistical significance was set at *P* < 0.05.

## Results

### Characteristics of participants

3,185 participants were included, representing a weighted population of 36,932,058. Among them, 49.97% were male and 50.03% were female. The average age was 40.00 (29.00, 50.00) years, and the weighted prevalence of sarcopenia was 7.84%. Participants were categorized based on CMI tertiles. Compared to those in the lowest CMI tertile, participants in the highest CMI tertile were generally older, more likely to be male, smokers, Mexican American, and had a higher likelihood of exhibiting abnormal BMI, hypertension, diabetes, CVD, and sarcopenia. They also had lower education, family income, and HDL-C levels but higher total dietary energy intake, WC, TG, TC, ALT, AST, BUN, SCR, SUA, and white blood cells (all *P* < 0.05). No significant differences were observed in sedentary time, vigorous activity, or CPK levels (all *P >* 0.05) (Table 1).

**Table 1:** Weighted baseline characteristics by CMI tertile groups (NHANES 2011-2018).

Characteristics	Overall	Tertile 1	Tertile 2	Tertile 3	*P*-value
	Weighted N = 36,932,058 Unweighted n = 3,185	Weighted N = 11,707,865 Unweighted n = 1062	Weighted N = 12,881,164 Unweighted n = 1061	Weighted N = 12,343,028 Unweighted n = 1062	
Age (years)	40.00 (29.00, 50.00)	35.00 (25.00, 47.00)	41.00 (31.00, 51.00)	44.00 (33.00, 53.00)	**<0.001**
Gender (%)					**<0.001**
Male	49.97	36.92	48.92	63.45	
Female	50.03	63.08	51.08	36.55	
Race/ethnicity (%)					**<0.001**
Mexican American	10.72	7.04	10.61	14.32	
Other Hispanic	7.74	6.12	8.11	8.90	
Non-Hispanic White	62.34	60.48	63.21	63.20	
Non-Hispanic Black	10.05	14.17	10.23	5.94	
Other Races	9.15	12.18	7.84	7.64	
Education (%)					**0.005**
Less than high school	13.47	9.67	13.73	16.80	
High school	21.85	21.00	21.03	23.51	
More than high school	64.68	69.32	65.24	59.68	
Marital status (%)					**0.014**
Married	51.86	49.78	52.84	52.80	
Widowed	1.70	1.61	1.15	2.36	
Divorced	9.26	6.36	9.61	11.64	
separated	2.47	1.95	2.57	2.84	
Never married	24.68	30.54	23.61	20.24	
Living with partner	10.04	9.75	10.21	10.12	
PIR (%)					**0.010**
<1.3	26.13	25.69	23.90	28.86	
≥1.3, < 3.5	34.43	31.94	35.59	35.59	
≥3.5	39.44	42.37	40.51	35.55	
BMI (%)					**<0.001**
Normal	28.91	54.62	24.17	9.48	
Overweight	33.48	28.86	38.49	32.65	
Obesity	35.95	12.66	36.24	57.74	
Sedentary time (%)					0.808
<3h	11.20	12.25	10.90	10.52	
≥3h, < 6h	32.87	33.08	33.41	32.11	
≥6h	55.93	54.67	55.69	57.37	
Vigorous activity (%)					0.128
No	76.01	78.64	76.10	73.44	
Yes	23.99	21.36	23.90	26.56	
Smokers (%)					**<0.001**
No	56.83	64.47	57.64	48.73	
Yes	43.17	35.53	42.36	51.27	
Hypertension (%)					**<0.001**
No	60.15	76.53	60.56	44.20	
Yes	39.85	23.47	39.44	55.80	
Diabetes (%)					**<0.001**
No	85.99	95.45	89.40	73.46	
Yes	14.01	4.55	10.60	26.54	
CVD (%)					**0.036**
No	96.08	97.90	95.46	95.01	
Yes	3.92	2.10	4.54	4.99	
Sarcopenia (%)					**<0.001**
No	92.16	96.69	92.52	87.50	
Yes	7.84	3.31	7.48	12.50	
Total energy intake (kcal)	2045.00 (1592.50, 2583.00)	1984.00 (1579.00, 2510.50)	2017.50 (1602.00, 2533.00)	2136.50 (1616.50, 2671.50)	**0.049**
WC (cm)	96.20 (86.20, 107.20)	84.90 (77.80, 93.80)	96.60 (88.80, 106.50)	106.10 (97.20, 117.20)	**<0.001**
TG (mmol/L)	1.12 (0.77, 1.69)	0.66 (0.51, 0.79)	1.11 (0.93, 1.31)	2.01 (1.60, 2.66)	**<0.001**
TC (mmol/L)	4.89 (4.22, 5.61)	4.58 (4.01, 5.28)	4.91 (4.29, 5.53)	5.17 (4.47, 6.05)	**<0.001**
HDL-C (mmol/L)	1.29 (1.09, 1.58)	1.63 (1.42, 1.91)	1.32 (1.16, 1.50)	1.06 (0.91, 1.19)	**<0.001**
ALT (U/L)	22.00 (17.00, 31.00)	18.00 (15.00, 23.00)	21.00 (17.00, 28.00)	28.00 (21.00, 39.00)	**<0.001**
AST (U/L)	22.00 (19.00, 27.00)	21.00 (18.00, 25.00)	22.00 (19.00, 27.00)	24.00 (20.00, 30.00)	**0.001**
BUN (mmol/L)	4.28 (3.57, 5.36)	4.28 (3.57, 5.00)	4.38 (3.25, 5.36)	4.51 (3.17, 6.36)	**0.007**
SCR (µmol/L)	72.49 (61.00, 84.86)	69.84 (60.11, 81.33)	72.49 (62.76, 84.86)	75.14 (62.76, 86.63)	**0.005**
CPK (IU/L)	103.00 (72.00, 164.00)	97.00 (67.00, 150.00)	102.00 (74.00, 166.00)	108.00 (77.00, 173.00)	0.680
SUA (µmol/L)	315.20 (261.70, 374.70)	279.60 (237.90, 333.10)	321.20 (273.60, 368.80)	356.90 (303.30, 404.50)	**<0.001**
Serum iron (µmol/L)	15.20 (11.50, 19.70)	15.00 (11.50, 19.50)	15.60 (11.60, 20.40)	14.90 (11.50, 19.20)	**0.002**
White blood cell (1000 cells/µL)	6.60 (5.60, 7.90)	5.90 (5.00, 7.10)	6.50 (5.60, 7.90)	7.10 (6.10, 8.60)	**<0.001**

Tertile 1: 0.04 ≤ CMI < 0.33; Tertile 2: 0.33 ≤ CMI < 0.71; Tertile 3: 0.71 ≤ CMI < 16.09.

Median (IQR) for continuous variables; % for categorical variables.

CMI, cardiometabolic index; PIR, poverty income ratio; WC, waist circumference; BMI, body mass index; TC, total cholesterol; TG, triglyceride; CVD, cardiovascular disease; HDL-C, high-density lipoprotein cholesterol; AST, aspartate transaminase; ALT, alanine transaminase; BUN, blood urea nitrogen; SUA, serum uric acid; CPK, creatine phosphokinase; SCR, serum creatinine.

### Factors associated with sarcopenia

Univariate regression analysis showed that sarcopenia was positively associated with age, hypertension, diabetes, CVD, BMI, total energy intake, WC, TG, TC, CMI, ALT, SUA, and white blood cell counts (all *P* < 0.05) (Table 2). In contrast, it was negatively associated with race, education, PIR, HDL-C, SCR, and CPK (all P < 0.05). Specifically, individuals with hypertension had a 0.872-fold increased risk of sarcopenia compared to those without hypertension (OR: 1.872; 95% CI: 1.462–2.398). Those with diabetes had a 1.699-fold increased risk (OR: 2.699; 95% CI: 2.060–3.537), and individuals with CVD had a 2.177-fold increased risk (OR: 3.177; 95% CI: 2.048–4.928). Regarding race, the risk of sarcopenia was lower in Other Hispanic, Non-Hispanic White, Non-Hispanic Black, and Other Races compared to Mexican Americans, with ORs of 0.669, 0.360, 0.120, and 0.348, respectively. For education, individuals with a high school education had a 43.4% lower risk of sarcopenia compared to those with less than a high school education (OR: 0.566; 95% CI: 0.401–0.799), while those with more than a high school education had a 61.2% lower risk (OR: 0.388; 95% CI: 0.291–0.519). Compared to participants with a PIR < 1.3, those in higher PIR groups had a decreased risk of sarcopenia, with ORs of 0.719 and 0.456.

**Table 2:** Univariate logistics regression analysis.

Variable	OR (95% CI)	*P*-value
Age	1.045 (1.033, 1.056)	**<0.001**
Gender		
Male	Ref	Ref
Female	1.041 (0.814, 1.332)	0.747
Race/ethnicity		
Mexican American	Ref	Ref
Other Hispanic	0.669 (0.454, 0.984)	**0.041**
Non-Hispanic White	0.360 (0.262, 0.494)	**<0.001**
Non-Hispanic Black	0.120 (0.070, 0.208)	**<0.001**
Other Races	0.348 (0.233, 0.519)	**<0.001**
Education		
Less than high school	Ref	Ref
High school	0.566 (0.401, 0.799)	**0.001**
More than high school	0.388 (0.291, 0.519)	**<0.001**
Marital status		
Married	Ref	Ref
Widowed	1.034 (0.437, 2.444)	0.939
Divorced	0.917 (0.584, 1.440)	0.708
separated	1.024 (0.552, 1.903)	0.939
Never married	0.659 (0.479, 0.908)	**0.011**
Living with partner	0.711 (0.459, 1.104)	0.129
PIR		
<1.3	Ref	Ref
≥1.3, < 3.5	0.719 (0.543, 0.950)	**0.020**
≥3.5	0.456 (0.329, 0.634)	**<0.001**
Sedentary time		
<3h	Ref	Ref
≥3h, < 6h	0.993 (0.663, 1.487)	0.971
≥6h	0.941 (0.639, 1.385)	0.757
Vigorous activity		
No	Ref	Ref
Yes	0.994 (0.743, 1.329)	0.966
Smokers		
No	Ref	Ref
Yes	1.020 (0.794, 1.310)	0.875
Hypertension		
No	Ref	Ref
Yes	1.872 (1.462, 2.398)	**<0.001**
Diabetes		
No	Ref	Ref
Yes	2.699 (2.060, 3.537)	**<0.001**
CVD		
No	Ref	Ref
Yes	3.177 (2.048, 4.928)	**<0.001**
BMI	1.101 (0.084, 1.118)	**<0.001**
Total energy intake	1.010 (1.002, 1.024)	**<0.001**
WC	1.039 (1.032, 1.046)	**<0.001**
TG	1.265 (1.165, 1.373)	**<0.001**
TC	1.315 (1.178, 1.469)	**<0.001**
HDL-C	0.485 (0.342, 0.689)	**<0.001**
CMI	1.370 (1.247, 1.504)	**<0.001**
ALT	1.010 (1.005, 1.014)	**<0.001**
AST	1.003 (0.999, 1.007)	0.220
BUN	1.033 (0.965, 1.106)	0.346
SCR	0.973 (0.965, 0.981)	**<0.001**
CPK	0.998 (0.997, 1.000)	**0.008**
SUA	1.002 (1.000, 1.003)	**0.010**
Serum iron	1.000 (0.981, 1.018)	0.967
White blood cell	1.186 (1.125, 1.250)	**<0.001**

OR, odds ratio; CI, confidence interval.

CMI, cardiometabolic index; PIR, poverty income ratio; WC, waist circumference; BMI, body mass index; TC, total cholesterol; TG, triglyceride; CVD, cardiovascular disease; HDL-C, high-density lipoprotein cholesterol; AST, aspartate transaminase; ALT, alanine transaminase; BUN, blood urea nitrogen; SUA, serum uric acid; CPK, creatine phosphokinase; SCR, serum creatinine.

### Multivariable logistics regression analysis of the relationship between sarcopenia and CMI

**Fig 2:**
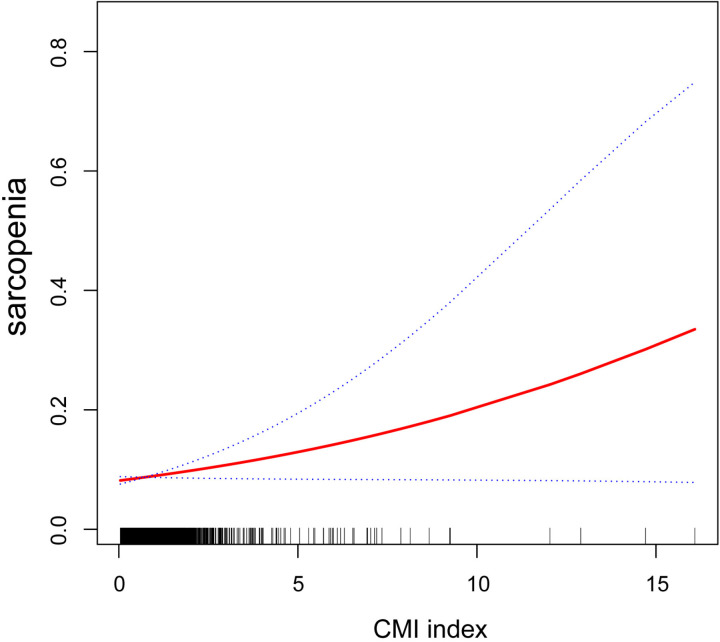
The smooth curve fitting plot of the association between CMI and sarcopenia.

A weighted logistic regression analysis revealed a positive association between sarcopenia and continuous CMI values and CMI tertiles (Table 3). In Model 3, after adjusting for all confounding variables, each one-unit increase in CMI (the smallest observable increment, unitless) was associated with a 12% higher risk of sarcopenia (OR: 1.12; 95% CI: 1.01–1.26). This positive correlation persisted to be significant even after converting CMI into tertiles. Participants in the second and third tertiles had 1.25 times (OR: 1.25; 95% CI: 0.79–1.97) and 1.68 times (OR: 1.68; 95% CI: 1.05–2.69) greater odds of sarcopenia, respectively, in comparison to those in the lowest tertile. The effect value progressively rose as the CMI levels grew (*P* for trend < 0.05). Through smooth curve fitting, we validated the linear association between the CMI and sarcopenia (Fig 2).

**Table 3:** Relationships between the CMI and sarcopenia risk.

Characteristics	Model 1 OR (95% CI)	*P*-value	Model 2 OR (95% CI)	*P*-value	Model 3 OR (95% CI)	*P*-value
**Total (n = 3209)**						
Continuous	1.37 (1.25, 1.50)	**<0.001**	1.30 (1.19, 1.44)	**<0.001**	1.12 (1.01, 1.26)	**0.038**
CMI Tertile						
Tertile 1	Reference		Reference		Reference	
Tertile 2	2.65 (1.75, 4.00)	**<0.001**	2.13 (1.40, 3.25)	**<0.001**	1.25 (0.79, 1.97)	0.334
Tertile 3	5.61 (3.82, 8.25)	**<0.001**	4.12 (2.76, 6.15)	**<0.001**	1.68 (1.05, 2.69)	**0.030**
*P* for trend		**<0.001**		**<0.001**		**0.018**

Model 1: Unadjusted variables

Model 2: Age, gender, race/ethnicity adjustments

Model 3: Adjusted for the following variables: age, gender, race/ethnicity, education level, marital status, PIR, BMI, hypertension, diabetes, CVD, total energy intake, sedentary time, smoking status, vigorous activity, TC, ALT, AST, BUN, SCR, CPK, SUA, serum iron, and white blood cell.

### Subgroup analysis

To further explore the relationship between sarcopenia and CMI more thoroughly across a number of subgroups, we conducted subgroup analyses and interaction tests based on sex, education level, PIR, BMI, sedentary time, vigorous activity, hypertension, diabetes, and CVD (Table 4). Subgroup analysis revealed that male participants (OR: 1.16; 95% CI: 1.03–1.31), individuals with a high school education (OR: 1.57; 95% CI: 1.06–2.33) or less (OR: 1.31; 95% CI: 1.11–1.54), individuals who were overweight (OR: 1.21; 95% CI: 1.03–1.56) or obese (OR: 1.52; 95% CI: 1.08, 2.11), those with a PIR < 1.3 (OR: 1.23; 95% CI: 1.03–1.47), and participants with sedentary time ≥ 3h but <6h (OR: 1.09; 95% CI: 1.01–1.16) or ≥ 6h (OR: 1.27; 95% CI: 1.06–1.53) were more likely to develop sarcopenia (all *P* < 0.05). In addition, individuals with hypertension (OR: 1.18; 95% CI: 1.03–1.36), diabetes (OR: 1.09; 95% CI: 1.02–1.38) and CVD diagnosis (OR: 2.51; 95% CI: 1.34–4.68) also exhibited an increased risk of sarcopenia (all *P* < 0.05).

**Table 4:** Subgroup analysis investigating the connection between sarcopenia and CMI.

Subgroup	OR (95% CI)	*P*-value	*P* for interaction
Gender			0.134
Male	1.16 (1.03, 1.31)	**0.015**	
Female	0.92 (0.69, 1.23)	0.569	
Education levels			**0.007**
Less than High School	1.31 (1.11, 1.54)	**0.001**	
High School	1.57 (1.06, 2.33)	**0.025**	
Above High School	0.92 (0.75, 1.14)	0.456	
PIR			0.351
<1.3	1.23 (1.03, 1.47)	**0.021**	
≥1.3, < 3.5	1.03 (0.85, 1.23)	0.792	
≥3.5	1.12 (0.80, 1.58)	0.506	
BMI			0.864
Normal	1.07 (0.94, 1.21)	0.683	
Overweight	1.21 (1.03, 1.56)	**0.005**	
Obesity	1.52 (1.08, 2.11)	**0.023**	
Sedentary time			**0.034**
<3h	0.63 (0.37, 1.10)	0.107	
≥3h, < 6h	1.09 (1.01, 1.16)	**0.011**	
≥6h	1.27 (1.06, 1.53)	**0.003**	
Vigorous activity			0.885
No	1.11 (0.95, 1.30)	0.202	
Yes	1.13 (0.96, 1.32)	0.131	
Hypertension			0.123
No	0.97 (0.78, 1.21)	0.800	
Yes	1.18 (1.03, 1.36)	**0.021**	
Diabetes			0.342
No	1.06 (0.89, 1.27)	0.511	
Yes	1.18 (1.02, 1.38)	**0.027**	
CVD			**0.003**
No	1.08 (0.96, 1.21)	0.219	
Yes	2.51 (1.34, 4.68)	**0.004**	

Adjusted for age, gender, education level, race/ethnicity, PIR, marital status, BMI, hypertension, CVD, diabetes, total energy intake, sedentary time, smoking status, vigorous activity, TC, ALT, AST, BUN, SCR, CPK, SUA, serum iron, and white blood cell except for subgroup variables.

The interaction analysis revealed significant differences in the subgroups of education level, sedentary time and CVD (all *P* for interaction < 0.05). These findings suggest that these factors influence the positive correlation between CMI and sarcopenia but not gender, PIR, BMI, vigorous activity, hypertension, or diabetes (all *P* for interaction > 0.05).

## Discussion

In this cross-sectional investigation involving a representative U.S. population, we found a significant correlation between sarcopenia and CMI. This correlation remained robust even after controlling for potential confounding variables. Our findings demonstrated substantial heterogeneity in the correlation between CMI and sarcopenia among subgroups based on education level, sedentary time, and the diagnosis of CVD. Specifically, higher CMI levels were linked to a higher incidence of sarcopenia in participants with long sedentary time and CVD diagnosis and those whose education level did not transcend high school.

In our study, we defined sarcopenia using the FNIH criteria, which utilizes the ASM to BMI ratio, distinguishing it from the EWGSOP2 definition, which employs the ASM to height^2^ ratio [26]. The FNIH criteria have been validated in multiple studies on sarcopenia and are widely used in clinical research [27–29]. BMI is considered a proxy for fat content, and normalizing ASM by BMI, rather than height², accounts for the influence of body fat on muscle mass. This approach is particularly relevant in populations with high obesity rates, such as U.S. adults, where adiposity plays a key role in muscle mass distribution. However, the choice of sarcopenia diagnostic criteria can affect the observed prevalence. For example, a study conducted in the Australian population found that the prevalence of sarcopenia was 12.9% when defined by FNIH criteria and 19.6% when defined by EWGSOP2 [30]. Similarly, in Chinese community-dwelling older adults, the EWGSOP2 definition resulted in a higher prevalence of sarcopenia (men: 6.5%, women: 3.3%) compared to the FNIH definition (men: 6.0%, women: 1.7%). Although the FNIH definition aligns with our focus on muscle mass adjusted for fat, this choice may limit the generalizability of our results to studies using other definitions. The lack of large-scale population studies comparing these standards means that future research should examine how different sarcopenia definitions impact risk associations across diverse populations.

Several studies have focused on the individual components of CMI and showed that elevated TG levels, reduced HDL-C, and WHtR are associated with a greater risk of sarcopenia and muscle loss [31–34]. Maintaining adequate skeletal muscle mass can help reduce specific lipids related to hyperlipidemia and cardiometabolic diseases [35]. Consistent with our results, our study demonstrates that higher levels of CMI were associated with an increased risk of sarcopenia. The CMI accounts for multiple metabolic disturbances and highlights their combined impact on sarcopenia risk. Integrating various markers of metabolic dysfunction into a single index provides a more holistic understanding of how these factors jointly contribute to muscle loss.

However, a study found that among young women, those who are underweight and have high HDL-C levels exhibit a higher prevalence of sarcopenia [36]. The finding that HDL-C shows significant differences only in females warrants further investigation in future research. The non-HDL cholesterol to HDL cholesterol ratio has emerged as a reliable indicator of lipid metabolism, which may serve as a valuable marker for monitoring and preventing sarcopenia, particularly in female cancer patients who appear more sensitive to these lipid-related changes [37]. Our research revealed that the positive correlation between CMI and sarcopenia was particularly pronounced in men, although the interaction test was not significant. The underlying reason may be that men generally have higher muscle mass and metabolic rates, making changes in metabolic-related indicators more pronounced in the presence of sarcopenia, which could raise the risk of metabolic syndrome [38]. For instance, testosterone levels in men are essential for both muscle mass and metabolism, and a decline in testosterone levels may exacerbate muscle loss and negatively impact metabolic health [39,40].

Elevated CMI reflects insulin resistance (IR), chronic inflammation, and ectopic fat deposition, contributing to muscle atrophy [41–43]. Insulin resistance impairs protein synthesis and promotes muscle breakdown, while visceral adiposity releases pro-inflammatory cytokines that accelerate muscle catabolism [42,44,45]. In sedentary individuals, physical inactivity exacerbates these metabolic disturbances, reducing muscle mass and function [46]. Additionally, individuals with CVD face heightened risks due to chronic systemic inflammation and oxidative stress, which impair mitochondrial function and muscle perfusion [47–50]. We found that CVD patients with high CMI have significantly greater odds of developing sarcopenia due to overlapping metabolic and vascular dysfunction pathways. Socioeconomic factors further compound these risks, as lower education levels are associated with limited health literacy, poor dietary habits, and reduced access to preventive care, all of which contribute to dyslipidemia, abdominal obesity, and ultimately higher CMI [51–53]. These disparities also limit engagement in resistance training or physical activity, accelerating muscle loss and exacerbating sarcopenia risk.

IR, a key feature of metabolic syndrome, reduces muscle cell sensitivity to insulin, impairing protein synthesis and promoting protein breakdown, leading to decreased muscle mass. IR disrupts insulin signalling pathways like the IRS-PI3K-AKT-mTOR axis and activates the ubiquitin-proteasome system to enhance protein degradation [54]. Increased lipolysis releases free fatty acids (FFAs), but excessive fat accumulation causes lipotoxicity, worsening IR and muscle mass reduction [55,56]. The buildup of harmful lipid intermediates, such as diacylglycerol and ceramide, contributes to muscle quality deterioration and mass reduction [55]. Chronic inflammation, often observed in dyslipidemia, also plays a significant role in muscle loss. Inflammatory cytokines like TNF-α and IL-6 activate signalling pathways in muscle cells, promoting protein degradation [57–59]. Inflammatory pathways, such as JNK and IKKβ, further induce IRS-1 serine phosphorylation, worsening insulin resistance [60,61]. Also, mitochondrial dysfunction is a common pathological mechanism linking insulin resistance and muscle atrophy. Mitochondria are essential for energy metabolism, and their dysfunction leads to reduced ATP production, impairing muscle cell function [62]. Mitochondrial impairment also elevates oxidative stress, further damaging muscle cells and accelerating degeneration [63]. These interconnected mechanisms—insulin resistance, chronic inflammation, and mitochondrial dysfunction—collectively contribute to muscle degradation in individuals with high CMI, highlighting the need for targeted interventions for sarcopenia.

This study offers several notable advantages. Firstly, it utilizes the CMI, a comprehensive marker integrating lipid profile indicators and obesity, providing a more holistic assessment of metabolic risk factors concerning sarcopenia. Secondly, the research draws on data from a large, well-established population, enhancing the generalizability and robustness of the findings. However, there are a few restrictions to consider. Firstly, the study’s cross-sectional design restricts our ability to establish causality between CMI and sarcopenia. Secondly, while we adjusted for several potential confounders, other factors such as medication use, dietary patterns, hormonal levels, or genetic predispositions were not included, which could influence the results. Additionally, it is essential to consider the potential for reverse causation, where sarcopenia could lead to changes in CMI components. For example, muscle loss may decrease physical activity [64], affecting metabolic health and increasing CMI values. Despite these limitations, the research offers novel perspectives on the connection between CMI and sarcopenia.

## Conclusion

This study further emphasizes the importance of metabolic factors in sarcopenia. By utilizing the CMI, we assessed its role in sarcopenia and identified a positive correlation between the risk of skeletal muscle loss and CMI. According to this research, CMI may be a valuable marker for sarcopenia, offering potential improvements in screening and management strategies for at-risk individuals. Nevertheless, to substantiate these findings and better understand the causal relationships between CMI and sarcopenia, further prospective studies are necessary. Such research will be crucial in validating our results and providing a more comprehensive exploration of how metabolic indices influence sarcopenia.

## Supporting information

S1 Table Definition of covariates. (DOCX)

S2 Table The collinearity assessment outcomes. (DOCX)
