# Supplementary material for: Association of the cardiometabolic index with sarcopenia among U.S. adults: NHANES 2011–2018 findings
Source: PLoS One. 2025 May 15;20(5):e0323905. doi: 10.1371/journal.pone.0323905 (PMC12080805; doi:10.1371/journal.pone.0323905)
Supplement: S1 Table — (DOCX) [file pone.0323905.s001.docx]

| S1 Table. Definition of covariates | |
| --- | --- |
| Covariates | Definition |
| BMI |  |
| Normal | 18.5–25 kg/m² |
| Overweight | 25–30 kg/m² |
| Obesity | ≥30 kg/m² |
| Smokers |  |
| Yes | Smoked at least 100 cigarettes in life |
| No | smoked less than 100 cigarettes in life |
| Hypertension | self-reported medical diagnosis; a mean diastolic blood pressure (DBP) of at least 80 mmHg or a mean systolic blood pressure (SBP) of at least 130 mmHg; taking a prescription for hypertension. |
| Diabetes | fasting blood glucose level ≥7.0 mmol/L, HbA1c ≥6.5%, oral glucose tolerance test 2-hour blood glucose level ≥11.1 mmol/L, or “yes” to the questions “Have you ever been told by a doctor or health professional that you have diabetes?” or “Are you now taking diabetic pills to lower blood sugar?” |
| CVD | CVD diagnosis was determined through the medical conditions questionnaire conducted during the interviews. The questionnaire included confirmation by a physician or other healthcare provider of congestive heart failure, coronary heart disease, angina, heart attack, or stroke. People with CVD were defined as those who responded “yes” to any of these questions. Those who gave a “no” or “don’t know” response or did not reply were excluded. |
| Vigorous work activity status | work involves vigorous-intensity activity that causes large increases in breathing or heart rate like carrying or lifting heavy loads, digging or construction work for at least 10 minutes continuously |
