# Supplementary material for: Association of the cardiometabolic index with sarcopenia among U.S. adults: NHANES 2011–2018 findings
Source: PLoS One. 2025 May 15;20(5):e0323905. doi: 10.1371/journal.pone.0323905 (PMC12080805; doi:10.1371/journal.pone.0323905)
Supplement: S2 Table — (DOCX) [file pone.0323905.s002.docx]

| S2 Table. The collinearity assessment outcomes | | | |
| --- | --- | --- | --- |
| variables | tolerance | VIF |  |
| Age | 0.604 | 1.654 |  |
| Gender | 0.547 | 1.829 |  |
| Race/ethnicity | 0.797 | 1.254 |  |
| Education | 0.736 | 1.359 |  |
| Marital status | 0.812 | 1.232 |  |
| Poverty–income ratio | 0.739 | 1.353 |  |
| Body mass index | 0.726 | 1.377 |  |
| Vigorous work activity status | 0.896 | 1.117 |  |
| Sedentary time | 0.877 | 1.140 |  |
| Smoking status | 0.861 | 1.162 |  |
| Hypertension | 0.758 | 1.320 |  |
| Diabetes | 0.814 | 1.229 |  |
| Cardiovascular disease | 0.929 | 1.076 |  |
| Total energy intake | 0.849 | 1.178 |  |
| Total cholesterol | 0.873 | 1.145 |  |
| Alanine transaminase | 0.407 | 2.459 |  |
| Aspartate transaminase | 0.443 | 2.257 |  |
| Blood urea nitrogen | 0.726 | 1.377 |  |
| Serum creatinine | 0.716 | 1.396 |  |
| Serum iron | 0.861 | 1.161 |  |
| Creatine phosphokinase | 0.878 | 1.139 |  |
| Serum uric acid | 0.609 | 1.641 |  |
| White blood cell | 0.861 | 1.161 |  |
